# Natural Products for the Treatment of Trachoma and *Chlamydia trachomatis*

**DOI:** 10.3390/molecules20034180

**Published:** 2015-03-05

**Authors:** Michael G. Potroz, Nam-Joon Cho

**Affiliations:** 1 School of Materials Science and Engineering, Nanyang Technological University, 50 Nanyang Avenue, Singapore 639798, Singapore; E-Mail: michaelg002@e.ntu.edu.sg; 2 Centre for Biomimetic Sensor Science, 50 Nanyang Drive, Singapore 637553, Singapore

**Keywords:** trachoma, *Chlamydia trachomatis*, natural products, antibacterial

## Abstract

The neglected tropical disease (NTD) trachoma is currently the leading cause of eye disease in the world, and the pathogenic bacteria causing this condition, *Chlamydia trachomatis*, is also the most common sexually transmitted pathogenic bacterium. Although the serovars of this bacterial species typically vary between ocular and genital infections there is a clear connection between genital *C. trachomatis* infections and the development of trachoma in infants, such that the solutions to these infections are closely related. It is the unique life cycle of the *C. trachomatis* bacteria which primarily leads to chronic infections and challenges in treatment using conventional antibiotics. This life cycle involves stages of infective elementary bodies (EBs) and reproductive reticulate bodies (RBs). Most antibiotics only target the reproductive RBs and this often leads to the need for prolonged therapy which facilitates the development of drug resistant pathogens. It is through combining several compounds to obtain multiple antimicrobial mechanisms that we are most likely to develop a reliable means to address all these issues. Traditional and ethnobotanical medicine provides valuable resources for the development of novel formulations and treatment regimes based on synergistic and multi-compound therapy. In this review we intend to summarize the existing literature on the application of natural compounds for controlling trachoma and inhibiting chlamydial bacteria and explore the potential for the development of new treatment modalities.

## 1. Introduction

### 1.1. Neglected Tropical Diseases

Neglected tropical diseases (NTDs) are a group of 17 major diseases that typically afflict the world’s poorest people [1]. These diseases are characterized by several common features: they usually occur in settings of poverty, they are chronic conditions, they often disable rather than kill, they have existed for centuries, and they promote poverty through their debilitating effects [2]. Due to the complex social and economic factors related to these conditions, plans have been developed to address these issues as a result of dialogue between private, public, and international organizations, the pharmaceutical industry, and national ministries of health [3]. However, it is important to note that one of the major challenges of treating these diseases is related to their tendency to develop into chronic conditions which are difficult to completely eradicate, even when using standard modern pharmaceuticals. One potential solution to overcome this complex problem is to identify and develop new means for effectively preventing, treating, and eradicating these chronic infections. For this process it is important to explore the natural remedies which are used in traditional medicines. It is likely that these hold important clues to groups of compounds which may be extracted and standardized into affordable and effective solutions to these challenging conditions. There are three NTDs classified as bacterial infections: leprosy, Buruli ulcer, and trachoma. Although, these particular diseases all have standard protocols for treatment with conventional antibiotics [3], even through the use of modern pharmaceuticals, complete eradication of these chronic conditions is problematic [4,5]. 

### 1.2. Trachoma and Chlamydia trachomatis

Trachoma is a bacterial infection caused by the Gram-negative bacterium *Chlamydia trachomatis* of the bacterial Phylum Chlamydiae. It is found primarily in Sub-Saharan Africa, the Middle East and North Africa [3] as well as to a lesser extent in Asia, Central and South America, and Australia. The symptoms are internally scarred eyelids, followed by eyelids turning inward, and if untreated this condition leads to the formation of irreversible corneal opacities and blindness [6]. As an eye infection, trachoma is one of the earliest recorded eye diseases, having been identified by the 27th century BC [6,7]. According to the WHO, although it is preventable, it is currently the leading cause of eye disease in the world, causing blindness in approximately 6 million and affecting the health of over 400 million worldwide [7,8]. 

Although trachoma as an NTD typically refers to the eye disease manifestation of a *C. trachomatis* infection, it is important to consider the role of this pathogenic bacteria as a sexually transmitted disease and the impact this has on the transmission of this bacteria. As a sexually transmitted disease, *C. trachomatis* infection is one of the most prevalent in the world [9,10], and the most commonly sexually transmitted pathogenic bacterium [11]. According to the WHO, there are an estimated 92 million new cases worldwide each year [9]. Chlamydial urogenital infections can cause a range of conditions such as cervicitis, urethritis, pelvic inflammatory disease and infertility [9]. The symptomatic or asymptomatic nature of these infections is of significant concern as it is estimated that approximately 75% of chlamydial infections in women and 50% in men are asymptomatic and therefore remain undetected and untreated [12]. In women, these undetected infections can cause severe permanent damage to the female genital tract [11,13] and may be passed on to newborns, this being a significant source of ocular trachoma infections in children. The transmission rate of *C. trachomatis* from mothers with chlamydial cervicitis to newborns is between 50% to 70% [14]. In infants, *C. trachomatis* may cause a range of conditions, such as endocervicitis, urethritis, conjunctivitis and afebrile pneumonia [15], and *C. trachomatis* transmission results in newborn conjunctivitis between 20% and 50% of the time and newborn pneumonia between 5% and 30% of the time [14]. These statistics highlight the connection between genital and ocular chlamydial serovars, as well as the various species of chlamydiae, such as, *C. trachomatis* and *C. pneumoniae*, and the infections they cause. 

#### 1.2.1. Chlamydial Developmental Cycle

**Figure 1:**
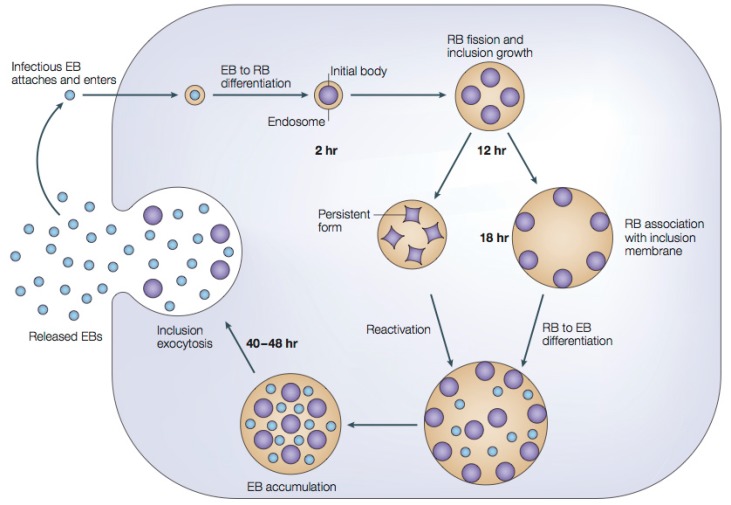
The developmental cycle of *Chlamydia trachomatis*. An infectious elementary body (EB) attaches and enters a eukaryotic host cell. The EB is surrounded by an endosomal membrane to form an inclusion. Within the inclusion, the EB differentiates into an active reticulate body (RB), which then divides by binary fission. Within 40–48 h the RBs transform back into infective EBs which are released to infect neighboring cells. If growth inhibitors are present during this process, intracellular C. trachomatis bacteria develop a non-replicating persistent form, which reverts to an infectious form upon removal of the inhibitor. Adapted with permission from [16]. Copyright 2005 Nature Publishing Group.

The various chlamydial species are known to cause persistent infections and are associated with a wide range of diverse chronic diseases such as trachoma, infertility, and coronary heart disease (CHD) [4,13]. A concern is that chlamydial infections may remain undetected and develop slowly over the course of tens of years with increasing severity [4]. A significant factor in the chronic nature of these infections is related to the unique developmental life cycle of chlamydial bacteria (Figure 1). Chlamydiae are obligate intracellular Gram-negative bacteria which require an eukaryotic host cell to reproduce within. Their developmental life cycle alternates over a 48 h period [16] between an infective metabolically inactive elementary body (EB) and a metabolically active reproductive reticulate body (RB) [4,9]. The elementary body (EB) form is infectious, but non-metabolically active and has adapted to exist in extracellular secretions [12,17]. These EBs attach to a eukaryotic host cell and are subsequently phagocytosed, wherein they reorganize into the reproductive reticulate body (RB) form [12]. The RB form exists and multiplies inside a membrane-bound vacuole known as an inclusion within the host cell cytoplasm [18]. In this state the RB can successfully inhibit phagolysosome formation and is able to utilize metabolic intermediates synthesized by the host cell [18]. The RBs divide asynchronously by binary fission and recondense to form EBs after a period of 30 to 48 h. Upon the formation of new EBs, the host cell lyses and the EBs are released so as to spread and infect new cells [12]. The EBs may spread through various bodily fluids, such as eye or nose discharge, or genital secretions of infected individuals [17]. Having these two developmental forms provides innate defenses against natural cellular responses to infection, and against standard therapeutic intervention [18]. 

#### 1.2.2. Conventional Chlamydial Treatment

The standard treatment for trachoma is with antibiotics and the most preferred treatment is a single oral dose of azithromycin (20 mg/kg, to a maximum dose of 1 g in adults) [3,19,20] or topical tetracycline (one percent eye ointment twice a day for six weeks) [19,20]. For chlamydial infections in general, both azithromycin and doxycycline are recommended by the US Centers for Disease Control and Prevention (CDC) and have a >95% microbiological cure rate [9]. Various antibiotics from different groups such as tetracyclines, macrolides, and fluoroquinolones have been shown to be effective against both *C. trachomatis* and *C. pneumoniae* [15]. However, it has been shown that although various chlamydial infections can be successfully treated with a range of antibiotics, eradication of chronic infections is difficult [4,5]. Due to the potential need for prolonged therapy there is an increased chance for the development of drug resistant chlamydial strains or other pathogenic organisms [18]. In several clinical studies *C. trachomatis* has been reported to exhibit both antibiotic resistance and persistence [9]. In addition, the related chlamydiae, *C. pneumoniae* has been recovered from cell cultures even after 30-day antibiotic treatment, has exhibited persistence in animal models after monotherapy, and after prolonged antibiotic treatment no effect was observed on *C. pneumoniae* antibody titers in large-scale trials [4].

With conventional antibiotic treatments there is difficulty in achieving the complete eradication of chronic chlamydial infections, the possibility of developing drug resistance, and the unintended creation of other antibiotic-resistant pathogens. A key issue is that most antibiotics target the metabolically active chlamydial reticulate bodies (RBs) through the inhibition of protein or nucleic acid synthesis, and have no effect on the metabolically inactive chlamydial EBs [4,18]. For this reason it is important to consider new compounds exhibiting various means of antimicrobial activity and finding novel nontoxic compounds for the treatment of chlamydial infections remains an important goal.

### 1.3. Drug Resistance and Biopharmaceuticals

Many conventional modern antibiotics are natural compounds obtained from microbial sources [21], with perhaps the most well-known being penicillin. This highlights the natural evolutionary state of bacteria living in complex microbial communities where organisms may produce either or both of offensive and defensive compounds [22] in the ongoing struggle for survival. Regarding the issue of drug resistance and the development of reliable treatments, it is necessary to consider both innate drug resistance, as well as drug resistance which has been acquired due to the use of clinical therapeutics. Innate drug resistance is the result of the natural evolutionary process of survival of the fittest [22,23] and a recent study by Wright *et al.* indicated that innate bacterial multi-drug resistance to known antibiotics may be relatively common [24]. In relation to acquired drug resistance, it is clear that resistance to natural antibiotics can develop within a few years, such as was the case for the antibiotics streptomycin, penicillin, and methicillin [22]. Drug resistance is now also being observed for even more advanced modern designer antibiotics, such as fluoroquinolones (FQs). As such, it has been suggested that as long as resistance is biochemically possible, it will occur [22], regardless of whether antibiotic compounds are synthetic or natural. The standard modern solution to this ongoing problem is the introduction of multi-drug therapy (MDT) [25], where combinations of several antibiotics are employed to overcome the defence mechanisms of resistant bacteria.

Natural biological materials and phytochemicals offer a wide range of compounds, such as flavonoids, coumarins, tannins, terpenoids, alkaloids, lipids, and peptides, many of which have been shown *in vitro* to exhibit antimicrobial as well as specific antichlamydial activity, and may prove to be sources of novel antichlamydial substances [18]. However, even as researchers move forward with the identification and isolation of novel single compounds from natural materials, acquired drug resistance may still develop. To progress, it is likely that even with new natural compounds it will be necessary to employ an MDT approach. It is also important to consider that traditional formulations are natural examples of MDT, and the modern concept of MDT may help to explain how some traditional herbal formulations may be successful in treating various bacterial infections. Drug synergies may occur through the use of several compounds which exhibit inhibitory effects through various mechanisms of actions (MOAs) [26], and this may also lead to a reduction in the rate of acquired drug resistance. However, it seems that the microbial battle which has been playing out in nature throughout the evolutionary process will continue, and it is unclear whether it will ever be possible to identify a permanent single compound solution to the treatment of specific bacterial infections.

## 2. Ethnobotanical & Traditional Medicine as Drug Leads

The World Health Organization has indicated that approximately 80% of the world’s population still relies primarily on plant-based traditional medicines for their primary healthcare needs [27]. It has also been estimated that 74% of pharmaceutically active plant derived compounds were discovered after investigations based on ethnobotanical traditions [27], and plant materials are now present in, or have provided models for approximately 50% of all modern pharmaceuticals [27]. Both trachoma and chlamydial infections have been commonplace throughout history and found in many parts of the world. In response to this, a wide range of botanical treatments have been developed to treat these conditions. By investigating traditional therapies, sources of potentially active compounds can be identified from ingredients, and in some cases, insight into basic chemical composition can be obtained through consideration of traditional extraction means and modes of delivery.

### 2.1. Traditional Medicine

Ethnobotanical and herbal medicine traditions provide a starting point for the identification of novel antichlamydial natural compounds and formulations. However, it should be noted that various resources, such as those discussed in the following section, often do not provide any specific data regarding the efficacy of these treatments, and provide only insights into species which may be of interest for further research.

In a 2011 study, 34 Bapedi traditional healers from the Limpopo province in South Africa were interviewed regarding treatments for sexually transmitted diseases, and chlamydia was one of four common diseases referred to. With regards to chlamydia, eight plant species used for treatment were identified: *Aloe marlothii subsp*. *marlothii*, *Eucomis pallidiflora subsp. pole-evansii*, *Gethyllis namaquensis (Schonland) Oberm.*, *Hypoxis obtusa Burch. ex Ker Gawl.*, *Kleinia longiflora DC.*, *Protea caffra subsp. caffra*, *Tribulus terrestris L.*, and *Ziziphus mucronata Willd.* [28]. Generally, root type material, in water based extractions, with oral delivery were the most common, and preparations commonly included more than one plant [28].

In a 2002 review of herbal medicines for sexually transmitted diseases, the natural compound berberine was identified as useful in the treatment of ocular *C. trachomatis* with the potential for the treatment of vaginal *C. trachomatis* infections [29]. Berberine is a quaternary ammonium salt from the protoberberine group of isoquinoline alkaloids and has a long history of use in China [30]. It is found in a variety of plants [30] and it has been indicated that tinctures, powdered dried root, or fluid and solid extracts of *Berberis aquifolium*, *Berberis vulgaris*, and *Hydrastis canadensis* can be used orally for treatment [29]. It was also indicated that vaginal chlamydial infections can be treated by local application of douches and vaginal depletion packs containing berberine [29], suggesting that this compound may also be effective when applied in topical formulations.

From ethnobotanical Arabic medicine, a boiled truffle water-extract from *Terfezia claveryi*, or desert truffles, is a traditional treatment for trachoma and various eye conditions [7]. Upon further investigation in 1981, Al-Marzooky [31] studied the *in vitro* antibacterial activity of truffle extracts and indicated that all aqueous, polar and non-polar extracts of *Terfezia claveryi* exhibited good antibacterial activity against *C. trachomatis*, as well as activity against a broad spectra of tested bacterial species [7]. A subsequent pilot clinical study, conducted by Al-Marzooky, indicated that *Terfezia claveryi* aqueous extracts were effective in the treatment of trachoma infected patients, although the treatment period required was longer than that of standard eye antibiotic treatments [7]. In support of these findings, studies from Chellal and Lukasova [32], and Janakat *et al.* [33,34], reported that compounds which were extracted from the desert truffles *Terfezia* and *Tirmania spp.* exhibited broad spectrum antibacterial activity. It was concluded that aqueous extracts of the truffle *Terfezia claveryi* contain a potent antimicrobial agent that is protein in nature [7]. Due to the complementary nature of these findings, it is possible that the proteins identified in these more recent studies were also a major factor in the antichlamydial activity observed earlier by Al-Marzooky, although further studies would be required to clarify this. Other plant species referred to for use in the treatment of trachoma include: *Abrus precatorius L.* [35], *Calpurnia aurea* [36], *Dodonaea viscosa Linn.* [37], *Erythrina abyssinica* [38], *Erythrina indica* [39], *Jatropha curcas* [40], *Primula auriculata* [41], and *Tinospora smilacina Benth.* [35].

### 2.2. Modern Herbal Formulations

There exist two clear examples with associated studies, representing the application of ethnobotanical traditions to the development of standardized herbal formulations. Both have demonstrated efficacy against urogenital chlamydial infections. This suggests that the active natural compounds in these formulations may prove to be effective against a range of chlamydial infections such as trachoma. These formulations provide potential leads for the identification and isolation of specific active compounds which may result in the development of standardized modern pharmaceuticals.

#### 2.2.1. Praneem Polyherbal Broad-Spectrum Antimicrobial Formulation

In 1995, Talwar *et al.* developed a natural polyherbal preparation with contraceptive and broad spectrum antimicrobial properties that was shown through Phase 1 clinical trials on humans to be effective in treating patients with vaginal *C. trachomatis* infections [42]. This formulation contained three active natural materials: purified extract from seeds of *Azadirachta indica* (neem), quinine hydrochloride, and saponins extracted from the pericarp of *Sapindus mukorossi*. These ingredients were suspended in a water-washable cream base for topical delivery. In addition to antichlamydial activity, this formulation has also been shown to exhibit antibacterial and antifungal action on *Gardnerella vaginalis* and *Candida albicans* [42]. Toxicity studies on rodents, rabbits, monkeys, and humans, were performed and this formulation was declared to be safe, with a lack of side effects, such as skin irritation or sensitization [42]. The efficacy and safety of this product were further verified by human trials which were conducted with the same primary ingredients in India, Brazil, Egypt, and the Dominican Republic which all produced similar results [29,42]. With regards to vaginal *C. trachomatis* infections, during a study in Safdarjung Hospital, New Delhi, infected subjects were asked to topically apply 5 mL of the cream every night for 14 days. By the 8th day, swabs taken from the subjects indicated that *C. trachomatis* had been cleared from the cervicovaginal region of every subject studied [42]. This indicates the effective antichlamydial properties of this formulation and supports the premise that similar results may be achieved across a range of chlamydial serovars. However, although this preparation has been shown to exhibit a broad range of antimicrobial properties and be particularly effective in treating *C. trachomatis*, the specific antichlamydial activity of this formulation is unclear and would require further studies to elucidate.

#### 2.2.2. BASANT Polyherbal Broad-Spectrum Antimicrobial Formulation

In support of this approach of combining a range of botanical materials based on established natural remedies, a more recent product was also developed in 2008. Bhengraj *et al.* also developed a natural polyherbal topical preparation, referred to as BASANT, with broad spectrum antimicrobial properties for the treatment of vaginal infections, which has been shown to exhibit antichlamydial activity *in vitro*. However, the ingredients used in this product were considerably different from the product developed by Talwar *et al.* in 1995. The 2008 formulation contained the four active natural compounds aloe vera, amla, curcumin, and reetha saponins. It should be noted that this combination of ingredients has also been shown to exhibit antimicrobial activity against *Neisseria gonorrhoeae*, *Candida spp*, HIV-1, and HPV-16 [9]. With regards to toxicity, BASANT has been shown to be free from local and systemic side effects, and exhibits no cytotoxic effect on the epithelial adenocarcinoma cells [9]. As a result of ongoing studies, these compounds were formulated into a cream and tested in Phase II clinical trials for treating the early stage of cervical dysplasia in women who tested positive for HPV-16 and -18 [9]. With regards to vaginal C. trachomatis infections, complete inhibition was observed in both pre-infection incubation and post-infection incubation assays with BASANT concentrations as low as 8 μg/mL [9]. In a later study, it was also determined that this combination of active ingredients could also be reformulated into a tablet form for vaginal insertion. *In vitro* studies using the tablet formulation indicated that antichlamydial activity against *C. trachomatis* is retained by the BASANT tablet [43]. As in the previous polyherbal product from 1995, although this formulation also exhibits broad spectrum antimicrobial properties, the mechanism of antichlamydial activity is not known and would require further studies to elucidate.

The ethnobotanical studies and traditionally inspired preparations discussed above provide potential natural sources for identifying and isolating specific novel compounds, as well as synergistic combinations of compounds exhibiting various forms of antichlamydial activity. From this perspective it is important to consider the kinds of compounds present in natural materials which are known to provide various biomedical effects and to begin to understand from what mechanisms are the antimicrobial effects achieved.

## 3. Biomedical Phytochemical Groups and Anti-Infective Action

There are diverse biochemical compounds which are present in natural materials and which have been shown to have applications in traditional and modern medicine. However, many of the medicinal effects of plants that are commonly discussed in terms of plant based biomedicine are derived from plant secondary metabolites. These can be categorized into the major phytochemical groups of quinones, flavonoids, polyphenols and tannins, coumarins, terpenoids and essential oils, alkaloids, lectins and polypeptides, glycosides, and saponins [27]. Each group is defined by a similar chemical structure and exhibits similar mechanisms of typical anti-infective activity [27]. It is important to note that these diverse secondary metabolites and other bioactive compounds exhibit a wide range of mechanisms for inhibiting microbial growth and that in complex natural materials there exist a plethora of compounds which may work synergistically together. It is these kinds of multi-compound formulations with singular compound synergies that have the potential for potent antimicrobial activity and a reduction in the development of microbial resistance.

With respect to plant based secondary metabolite compounds with antichlamydial effects the only studies available investigate the activity and properties of phenols and flavonoids. Of the various other bioactive compounds known to exhibit antimicrobial properties, lipids and peptides have also been investigated with respect to antichlamydial activity.

### 3.1. Phenols/Flavonoids

**Figure 2:**
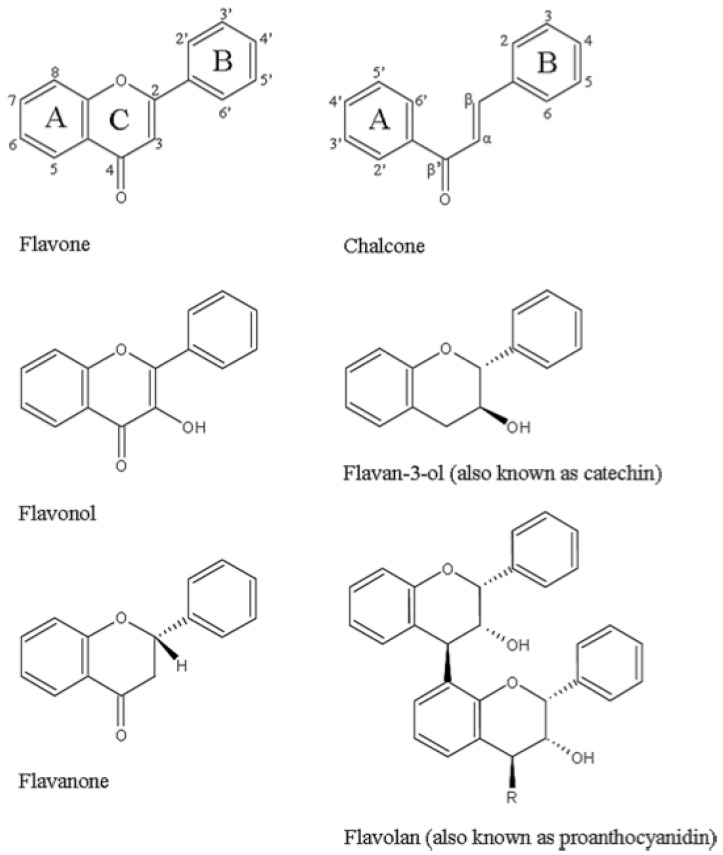
Structures of six of the main classes of antibacterial flavonoids, namely flavones, flavonols, flavanones, chalcones, flavan-3-ols, and flavolans. Adapted with permission from [45]. Copyright 2011 Elsevier B.V. and the International Society of Chemotherapy.

Due to the focus of existing studies with regards to the antichlamydial activity of specific phenols and flavonoids and the significant interest that these groups of compounds has received in recent years, this section will be covered in the most detail. Phenols and flavonoids comprise a very diverse group of compounds with several mechanisms identified to be responsible for the antimicrobial activity that they exhibit [44,45]. Flavonoids are a group of natural polyphenolic compounds which have been shown to possess a wide range of clinically relevant properties [8]. However, other biologically active phenolic compounds exist that are not classified as flavonoids. Flavonoids generally occur in all higher plants and can be considered safe for consumption as they are typically consumed daily in foods such as fruits, vegetables, and herbs, as well as plant-derived beverages, such as tea and wine [4,18]. Within plants they act as secondary metabolites to protect the plant against damage from radiation and pathogenic micro-organisms [44]. Overall, polyphenols are a large group of phytochemicals containing more than 8000 compounds [18]. Many of them contain a flavan nucleus comprising of 15 carbon atoms arranged in three rings (C6-C3-C6), and typically occur as glycosylated derivatives [4]. There are 14 subclasses of flavonoids which are defined due to specific structural arrangements such as positions of functional groups. Glycosylation may also occur at multiple sites with various saccharides and acylation of the saccharides [46]. This large range of compounds exhibits significant variation in chemical properties and solubility [46]. In terms of the identification of novel new therapeutics, these subgroups and patterns of glycosylation have also been shown to be closely associated with plant taxonomy [46]. The majority of reports identifying antibacterial properties of flavonoids refer to six of the 14 subclasses [45] and the skeleton structures of these six subclasses are shown in Figure 2.

The bioavailability of flavonoids varies greatly and they are typically eliminated from the body quite quickly [4]. This suggests that they should be ingested or added to the body regularly to maintain high concentrations [4]. However, it should be noted that some phenolic compounds accumulate inside tissues, resulting in higher concentrations compared to that found in plasma [4]. Flavonoids have been suggested to exhibit a wide range of effects, such as, antioxidant properties, modulation of drug-metabolising enzymes, modification of platelet aggregation, and activity related to the immune system, as well as antiviral and antibacterial activities [44]. This wide range of effects highlights the possibility that the apparent microbial inhibitory activity of polyphenolic compounds is related to both direct and indirect mechanisms. With regards to direct antibacterial activity, there are five mechanisms which have been proposed: cytoplasmic membrane damage, inhibition of nucleic acid synthesis, inhibition of energy metabolism, inhibition of cell wall synthesis, and inhibition of cell membrane synthesis [45]. In addition to direct antibacterial activity it has been suggested that flavonoids interfere with various bacterial virulence factors, including enzymes, toxins, and signal receptors [45].

There are several studies investigating the antibacterial activity of specific phenolic and flavonoid compounds with regards to chlamydiae such as *C. trachomatis* and *C. pneumoniae*. In 2003, Yamazaki *et al.* investigated the *in vitro* inhibitory effects of tea polyphenols on the proliferation of *C. trachomatis* and *C. pneumoniae* [15]. In 2005, Törmäkangas *et al.* investigated the *in vivo* treatment of acute *C. pneumoniae* infection with the flavonoids quercetin and luteolin and an alkyl gallate, octyl gallate, in a mouse model [44], based on the results of preliminary studies into natural products in the process of finding new drug candidates by Vuorela *et al.* in 2004 [18]. In 2006, Alvesalo *et al.* investigated the inhibitory effect of dietary phenolic compounds on *C. pneumoniae* in cell cultures [4]. In 2009, Hao *et al.* investigated the potential of the flavonoid baicalin to suppress the expression of chlamydia protease-like activity factor in Hep-2 cells infected by *C. trachomatis* [8]. 

#### 3.1.1. Catechins—Membrane Disruption

Catechins are a type of flavan-3-ol and are ubiquitous constituents of vascular plants, and frequent components of traditional herbal remedies. The tea polyphenol extract studied by Yamazaki *et al.* comprised mainly catechins and exhibited an *in vitro* inhibitory effect on both *C. trachomatis* and *C. pneumoniae*. However, the inhibitory concentrations required were relatively high compared to the MICs of antibiotics such as tetracyclines [15]. It has been indicated that catechins damage [47] or disrupt the permeability [27] of lipid bilayers and therefore it is likely that the antibacterial properties observed in this study were due to the mechanism of cytoplasmic membrane damage. This direct antibacterial mechanism supports the broad spectrum antimicrobial properties observed in other studies where tea polyphenols have been shown to inhibit influenza virus [48,49], *Vibrio cholerae* [50], *Staphylococcus aureus* [51], *Campylobacter jejuni*, *C. coli* [52], and others [15].

Yamazaki *et al.* went on to suggest that topical application of water based tea leaf extracts obtainable by boiling tea leaves in water may be adequate to achieve useful antichlamydial properties. This observation was based on the concentrations observed which were necessary to achieve inhibitory effects. The results of this study indicated complete inhibition of *C. trachomatis* for Polyphenon 70S extract concentrations of 0.4 mg/mL and above. This tea extract, comprised a range of tea polyphenols: epigallocatechin, epicatechin, epigallocatechin gallate, epicatechin gallate, and gallocatechin gallate. The dominant constituent being epigallocatechin gallate (EGCg) which is believed to be a major contributor to the observed anti-microbial effects [15]. It was proposed that from a simple extraction process it would be possible to produce an EGCg concentration of 2.1 to 2.4 mg/mL, which may be high enough to provide antichlamydial activity when used for washing infected regions [15].

#### 3.1.2. Luteolin—Increased Cellular Apoptosis

Luteolin is a flavone which is commonly found in a wide range of plants, such as trees, herbs, and vegetables. An *in vivo* study with mouse models carried out by Törmäkangas *et al.* showed that the flavonoid luteolin was able to suppress *C. pneumoniae* inflammation in lung tissue, the development of *C. pneumoniae*-specific antibodies, and the presence of chlamydia in lung tissue. This was an extension of preliminary antibacterial studies wherein luteolin was shown to inhibit *C. pneumoniae* in HL cell cultures [18]. With regards to the observed anti-inflammatory effect, it was noted that chlamydiae were still present in tissues even when inflammation was completely abolished. It was proposed that these reduced inflammatory responses may be due to a suppression of NF-kB-mediated cascades as suggested by other studies indicating that luteolin can inhibit phosphorylation cascades and proinflammatory cytokine and chemokine production either *in vitro* or in animal models [53,54]. However, it should be noted that if luteolin directly reduces the natural inflammatory process, then this may cause a reduction in the body’s immune response and a subsequent decrease in the immune system contribution to overall antichlamydial activity. The observed reduction in *C. pneumoniae*-specific antibodies found in serum also suggests a decrease in the natural immune response. Conversely, although luteolin may suppress natural immune responses, reductions of the presence of chlamydia in lung tissue were still observed and it has been suggested that luteolin induces cellular apoptosis via interference with the mitochondrial pathway [44,55]. This effect counteracts the reported the ability of *C. pneumoniae* to inhibit apoptosis of the host cell, which has also been attributed to interference of the mitochondrial pathway [44,56]. Therefore, it is proposed that luteolin negates the antiapoptotic effect of chlamydia which leads to apoptosis of the infected host cells. The resulting release of intracellular chlamydial particles would cause the chlamydial infection to become vulnerable to the hosts natural antimicrobial defenses [44].

#### 3.1.3. Baicalin—Improved Immune Detection

Baicalin is a flavone derived from Radix scutellariae, the dried root of the medicinal plant *Scutellariae baicalensis*, which is a medicinal plant traditionally used in Oriental medicine. It has been shown to exhibit a variety of effects, such as, antimicrobial and anti-inflammatory activities, and previous studies have indicated that it can inhibit several strains of *C. pneumoniae* both *in vitro* [57] and *in vivo* [58]. Hao *et al.* studied the ability of the flavonoid baicalin to block *C. trachomatis* infection *in vitro* and found that the baicalin successfully blocked the infection of Hep-2 cells. It was observed that baicalin was able to down-regulate the production of a chlamydia-secreted protein, chlamydia protease-like activity factor (CPAF) [7]. CPAF has been shown to degrade host transcription factors such as RFX5 which are required for major histocompatibility complex antigen expression. It has been suggested that this may allow chlamydia to escape efficient immune detection [59,60], which is an important defense mechanism for chlamydial bacteria. Therefore, by baicalin targeting and down-regulating the production of CPAF, the immune system may more effectively detect the chlamydial infection [8]. However, in relation to the inflammatory mechanisms of chlamydial infections, CPAF is synthesized as a proenzyme by chlamydiae and has to be processed into intramolecular dimers to acquire proteolytic activity. As such, it is through this process that CPAF also plays a critical role in inflammation induced by chlamydial infections [61,62]. In this regard, it is also important to note that the down regulation of CPAF may reduce host inflammation and lead to a decrease in natural immune response and subsequent decrease in the immune system contribution to overall antichlamydial activity. 

#### 3.1.4. Polyphenols—Antichlamydial Activity and Polyphenol Structure

**Figure 3:**
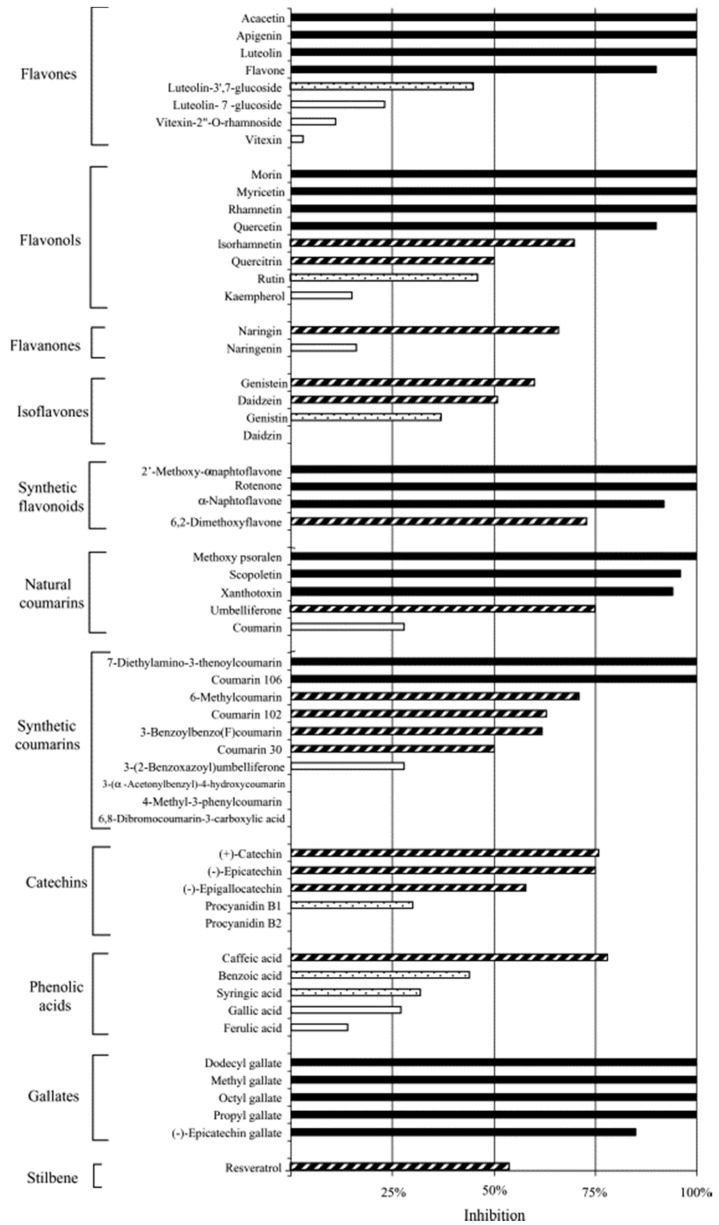
Average inhibition percentages of natural or natural-based polyphenols and synthetic derivatives against *C. pneumoniae* at 50 μM concentration (*n* = 4 or more). Categories of activity are determined to be: highly active (black bar) = 85%–100% inhibition compared to DMSO-controls; active (striped bar) = 50%–84%; moderately active (black dotted bar) = 30%–49%; inactive (white bar) = <30%. Adapted with permission from [4]. Copyright 2005 Elsevier Inc.

The most comprehensive study to date regarding the antichlamydial activity of flavonoids was conducted by Alvesalo *et al.* to determine the antichlamydial activity of 57 natural flavonoids and other natural polyphenols and structurally similar synthetic compounds against *C. pneumoniae* in a human cell line (HL). In terms of applicability to human treatment, it is important to note that all of the compounds were found to be non-toxic to host cells at the concentrations used in the study [4]. With regards to activity against *C. pneumoniae*, 37% (21/57) of the compounds were classified as highly active, 28% (16/57) active, 11% (6/57) moderately active, and 24% (14/57) inactive, as shown in Figure 3. The broad nature of the antichlamydial activity of polyphenols was highlighted by the fact that there were highly active compounds identified in 6 of the 11 groups studied. The most active group was that of gallates, with the groups of flavones, flavonols, synthetic flavonoids, and natural coumarins also showing typically high activity. In terms of compound synthesis, it is interesting to observe that although natural coumarins showed typically high activity, the group of synthetic coumarins had the greatest number of compounds with no activity [4]. Therefore, it is clear that modifications using these compounds as natural templates requires careful consideration.

In relation to the issues of flavonoid bioavailability and potentially low plasma concentrations, it was shown in this study that at least some of these compounds can accumulate inside cells or be attached to cell membranes [4]. Four phenolic compounds: quercetin, rhamnetin, morin, and octyl gallate, were studied, whereby HL cells were incubated for 24 h with each compound prior to infection with *C. pneumoniae*, after which time the cells were infected and then grown in infection medium without the test compound for 72 h. Although only being added during the pretreatment process, these compounds still decreased the infectivity of *C. pneumoniae* to 0%–50% of that in untreated controls. This clearly indicates the ability of these compounds to accumulate in cells or on cell membranes at levels high enough to maintain antimicrobial properties. However, when the compounds were present throughout the cell culturing process, infectivity was even lower, ranging from 0% to 32% [4].

Alvesalo *et al.* went on to consider the relationship between molecular structure and observed antichlamydial activity. At a fundamental level their study showed that a wide range of structurally different phenolic compounds may exhibit antichlamydial activity [4]. However, several more specific trends were also apparent. The presence of sugar moieties appears to be a major structural feature of significance. Both flavones and flavonols have the same basic structure and both groups contain active and inactive compounds, although upon more careful analysis, this variation in compound activity can be attributed to the presence of sugars. All of the compounds exhibiting over 70% antichlamydial inhibition are free from sugars, and all compounds with 50% inhibition or less contain one or more sugar moieties [4]. A more minor structural feature influencing activity relates to hydrophobicity and the presence of methoxy groups. The flavonols quercetin, rhamnetin, and morin have the same basic three-ring structure and have been shown to be able to penetrate phospholipid membranes [4]. However, while rhamnetin is able to completely block the production of infective chlamydial EBs, treatment with quercetin and morin result in inclusions being observed after repassage [4]. The only structural difference between rhamnetin and quercetin with the potential to explain this variation in antichlamydial activity is a methoxy group in the A-ring of rhamnetin which causes the molecule to be more hydrophobic [4]. Based on this comprehensive study, it seems likely that with more research, more clear connections between molecular structure and antimicrobial activity will become apparent.

Overall, it is clear that a wide range of flavonoids have been shown to exhibit antichlamydial activity. Of the chlamydial species referred to in these studies we can see that both *C. pneumoniae* and *C. trachomatis* are sensitive to catechins from tea extracts and that the flavone, baicalin, successfully inhibited *C. trachomatis* *in vitro* [15]. Also, the flavone luteolin and another 21 flavonoids were shown to be highly active at inhibiting *C. pneumoniae* [4,44]. From these studies it also becomes apparent that there are various mechanisms of antichlamydial activity. Flavan-3-ol catechins are proposed to primarily rupture cell membranes [15], whereas the flavones baicalin and luteolin exhibit indirect mechanisms in the form of the negation of the antiapoptotic effect of chlamydia [44] and improved pathogen identification [8]. Also, all of the four polyphenols tested, quercetin, rhamnetin, morin, and octyl gallate, have been shown to be able to accumulate within cells or attach to cell membranes such that their antimicrobial activity remains even when removed from surrounding culturing media [4]. The diversity of mechanisms and degree of inhibitory activity identified in these studies support the role of flavonoids in contributing to a multi-mechanism approach to the effective control and inhibition of a range of chlamydial infections.

### 3.2. Lipids/Fatty Acids

Lipids have long been known to exhibit a wide range of antimicrobial activity [63] and the studies relating to the antichlamydial activity of lipids directly investigate the inhibitory action of a range of fatty acids and monoglycerides. These studies indicate the potential for various lipidic compounds to be effectively utilized for controlling chlamydial infections [11,17] and highlight a common theme of membrane disruption as the primary mechanism of action as is clearly shown from electron micrographs from each of the studies.

#### 3.2.1. Fatty Acids and Monoglycerides—Membrane Disruption

**Figure 4:**
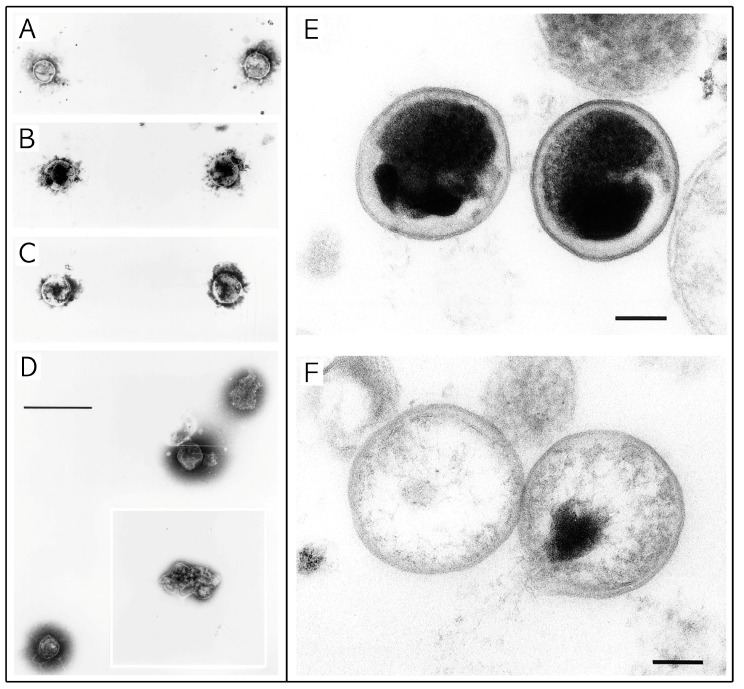
Electron micrographs of *C. trachomatis* with and without exposure to lipidic compounds. (**A**–**D**) Exposure of *C. trachomatis* EBs to monocaprin. The EBs were untreated (A) or treated with 10 mM monocaprin for 1 min (B), 5 min (C), and 10 min (D). After 10 min, the EBs appear deformed or disrupted (D, inset). Bars, 1 μm. (**E**,**F**) Exposure of *C. trachomatis* EBs to 1-*O*-hexyl-*sn*-glycerol. (E) *C. trachomatis* EBs exposed to sucrose-phosphate-glutamine buffer (SPG) only. (F) EBs exposed to 50 mM 1-*O*-hexyl-*sn*-glycerol for 90 min appear as hollow structures. Parts A to D are adapted with permission from [11]. Copyright 1998 American Society for Microbiology. Parts E and F are adapted with permission from [17]. Copyright 1998 American Society for Microbiology.

Bergsson *et al.* showed that *C. trachomatis* can be inactivated by exposure for 10 min to 10 mM (final concentration) lauric acid, a 12-carbon saturated fatty acid (12:0), and to capric acid (10:0) and its 1-monoglyceride, monocaprin. It is important to note that a range of other fatty acids and their monoglycerides were also studied and shown to exhibit negligible effect. These included caprylic acid (8:0), monocaprylin (8:0), monolaurin (12:0), myristic acid (14:0), palmitoleic acid (16:1), monopalmitolein (16:1), oleic acid (18:1), and monoolein (18:1) at concentrations of 20 mM (final concentration, 10 mM). This narrow range of closely related compounds which exhibit antichlamydial activity caused the authors to suggest that these lipids have specific antichlamydial effects. This is supported by other studies showing that the herpes simplex virus type 1 (HSV-1) is susceptible to a wider range of fatty acids and monoglycerides. Also, capric acid, which was shown to exhibit high antibacterial activity against *C. trachomatis*, exhibited no activity against HSV-1 under the same conditions [11], indicating that this antimicrobial activity is not necessarily broad spectrum in nature. It was further determined that this inhibitory activity was the result of permanent and irreversible damage to the *C. trachomatis* EBs. The EBs were exposed to and then removed from the lipid before inoculation into cell cultures, and after 5 min exposure, EB viability was shown to decrease, and after 10 min was completely lost [11]. This necessary inactivation time, of 5 to 10 min, was supported by electron microscope observations (Figure 4A–D), which showed no visible changes to EBs after 5 min. However, after 10 min the treated EBs appeared deformed and partially disintegrated (Figure 4D) [11]. The irreversible deactivation of *C. trachomatis* EBs combined with the observed cell rupture and disintegration after 10 min suggests that these lipids inactivate the bacteria by affecting and disrupting the outer membrane. This is supported by a previous study showing that linoleic acid disrupted both the viral envelope of vesicular stomatitis virus, as well as the cellular membrane on Vero cells [64]. 

In support of the potential for the production of specific antichlamydial formulations incorporating lipidic compounds, several pharmaceutical formulations containing monocaprin have already been developed and have been shown to be potent inactivators of *C. trachomatis*, HSV-2, and human immunodeficiency virus *in vitro* [11]. As a comparison of the large scale applicability of such lipidic compounds, the detergent nonoxynol-9 has also been widely studied and applied to formulations for the prevention of STDs even though it is known to be toxic in cell cultures and cause irritation of mucosal tissues with frequent application [11]. Monocaprin, however, has been shown to have a greater inhibitory effect against chlamydia and have a low level of toxicity *in vivo* at relevant concentrations [11].

#### 3.2.2. Synthetic Lipids—Membrane Disruption

Synthetic lipids have also been shown to have potential for use in antichlamydial formulations [17]. Lampe *et al.* studied the activity of five synthetic lipids derived from human breast milk. Natural compounds were isolated and modified so as to maintain antimicrobial activity while increasing their stability and solubility. The most active of these, 2-O-octyl-sn-glycerol, completely inhibited the growth of *C. trachomatis* when applied at a concentration of 7.5 mM for 120 min [17]. The remaining four compounds, 1-O-octyl-, 1-O-heptyl-, 2-O-hexyl-, and 1-O-hexyl-sn-glycerol, exhibited less activity. The observed antichlamydial activity of 2-O-octyl-sn-glycerol indicated the possibility that it also exhibits some specific antichlamydial effects, as in previous studies, all of these synthetic lipids were shown to have inhibitory activity against *Escherichia coli*, *Salmonella enteritidis*, and *Staphylococcus epidermidis* [65]. In consideration of developing topical microbicides for sexually transmitted diseases it was also determined that this lipid activity was unaffected by conditions commonly present in the vagina, such as, the presence of up to 10% human blood or alterations in pH from 4.0 to 8.0 [17]. In terms of toxicity, preliminary studies indicate that there is no vaginal irritation in rabbit models from lipid concentrations as high as 120 mM [17]. The antichlamydial properties were attributed to the disruption of the chlamydial lipid membrane. This process was observed by electron microscopy (Figure 4E,F) such that after EBs were exposed to 50 mM 1-O-hexyl-sn-glycerol for 90 min, the EBs appeared to be hollow shells (Figure 4F) with the cell membrane ruptured and the cytoplasmic contents having leaked out of the cell [17]. Furthermore, these synthetic lipids were determined to exhibit no cellular toxicity [17], which supports the direct disruption of the chlamydial lipid membrane as the reason for the observed inhibition of chlamydial growth, rather than damage to the host cells.

Various studies into the antimicrobial activity of lipids suggest a general theme of broad spectrum antimicrobial properties [66] as well as more specific antichlamydial activity of some compounds [11,17]. The high levels of antichlamydial activity combined with low levels of host cell toxicity and biologically relevant concentrations from both of these lipid studies suggests that both of natural and synthetic lipidic compounds may be appropriate for use in specific antichlamydial formulations.

### 3.3. Peptides

One remaining group of natural compounds that has been studied in direct relation to having antichlamydial properties is peptides. Peptides are short chains of amino acid monomers linked by peptide (amide) bonds [67]. A wide variety of peptides exist in both plants and animals and are known to exhibit broad spectrum antimicrobial activity [67]. The primary mode of antimicrobial action of many peptides is membrane disruption [67] which is in line with the only antichlamydial peptide study available.

#### Cecropin Peptides—Membrane Disruption

**Figure 5:**
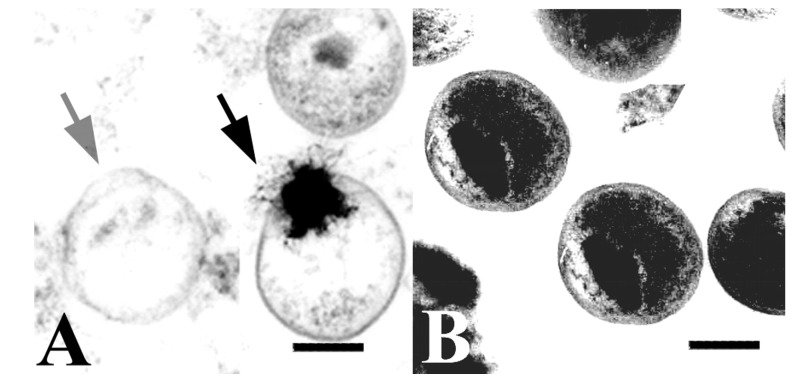
Electron micrographs of *C. trachomatis* exposed to cecropin peptide D2A21 for 90 min. (**A**) Organisms treated with D2A21 appear to be hollow or in the process of leaking their cytoplasmic contents (black arrow). (**B**) Untreated organisms incubated in SPG only. Bar = 0.5 μm. Adapted with permission from [12]. Copyright 2002 American Society for Microbiology.

In terms of antichlamydial activity, Ballweber *et al.* investigated whether two cecropin peptides, D2A21 and D4E1, exhibit antibacterial activity against *C. trachomatis*, and could be formulated into effective topical microbicides. Cecropin peptides are a group of antibacterial peptides that were initially identified in the pupae of the cecropia moth, and were subsequently identified in other insects (e.g., bactericidin, moricin, and sarcotoxin) as well as in pig intestines (cecropin P) and tunicates [68]. It was found that both peptides could completely inhibit *C. trachomatis* EBs *in vitro*. These peptides have also been shown to exhibit more broad spectrum *in vitro* activities against human immunodeficiency virus, *N. gonorrhoeae*, *Gardnerella vaginalis*, *Trichomonas vaginalis*, and *Candida albicans* [12]. However, when incorporated into a gel formulation, only the peptide, D2A21, was shown to inhibit *C. trachomatis* under conditions present in the vaginal vault and anogenital tract [12]. The observed antichlamydial properties of D2A21 have been attributed to membrane lysis or disruption. This was clearly observed using electron microscopy (Figure 5). After *C. trachomatis* EBs were exposed to the peptide D2A21 for 90 min, the cell membranes appear to have ruptured, and the cytoplasmic contents have leaked out, leaving empty hollow shells (Figure 5A) [12]. This is supported by previous studies indicating that the mode of action for this class of peptides is the creation of pores or channels through the bacterial membrane [69,70]. However, it must be considered that the antibacterial activity may also be more complex, as other peptides in this class have exhibited other antibacterial mechanisms, such as, the release of mitochondrial respiratory control, the inhibition of protein import, and the inhibition of bacterial respiration [68]. It is important to note that during postinoculation assays, D2A21 failed to inhibit *C. trachomatis*. This suggests that D2A21 does not penetrate eukaryotic cells [12], and consequently may not be effective against the reproductive reticulate body (RB) stage of the chlamydial life cycle.

In relation to microbicidal formulations, the *in vitro* results of this study indicate that both of the antimicrobial peptides, D2A21 and D4E1, are effective microbicides at concentrations achievable *in vivo*. However, the activities of these peptides are sensitive to pH conditions, which may or may not be alleviated upon their incorporation into a gel formulation [12]. An additional concern is that a gel formulation including 2% D2A21 demonstrated significant cytotoxicity to McCoy cells during *in vitro C. trachomatis* assays [12]. However, in general, cecropins have been shown to exhibit minimal cytotoxicity against mammalian cells [68,69], and therefore more studies regarding the efficacy and cytotoxicity of these products in humans should be undertaken. 

Although only the one study on antichlamydial peptides is available at this time, the extensive literature on the antimicrobial activity of peptides supports the membrane disrupting process [69] observed for cecropin peptides and chlamydia, and it seems likely that other peptides which exhibit antichlamydial activity may exist in plants, insects, or animals.

## 4. Perspectives

Based on the existing literature in this field it seems promising that with continued research it will be possible to formulate a treatment regime for trachoma and related chlamydial infections based on combinations of naturally derived compounds. Several formulations of commonly used medicinal plant species have exhibited antichlamydial activity both *in vitro* and *in vivo* as well as in clinical studies, although the specific compounds and mechanisms of action are yet to be determined [9,29,42,43]. Studies indicate that antichlamydial proteins may be extracted from a specific species of desert truffle [7], and that a naturally occurring antichlamydial alkaloid in the form of quaternary ammonium salt may be extracted from a range of plants [29]. Secondary plant metabolites such as polyphenolic flavonoids have been shown to exhibit antichlamydial activity through a number of means: direct membrane disruption [15]; improvements in pathogenic cell recognition [8]; and increased apoptosis of infected cells [8,44]. Based on studies of flavonoids with other bacteria [45,71] we can expect that they may also exhibit more diverse effects upon chlamydial cells as a more comprehensive range of studies is performed. Lipids and peptides have also been shown to hold the potential to provide antichlamydial activity through membrane interactions and disruption although exhibiting some toxicity toward host cells [11,12,17]. Furthermore, studies of plant species used in traditional ethnobotanical medicine suggest that there is a wide range of new natural materials yet to be explored [28].

Even considering the relatively small number of individual natural compounds reviewed in this paper it can be suggested that it is possible to both directly and indirectly target both of the chlamydial infective elementary bodies (EBs) and the reproductive reticulate bodies (RBs). By combining compounds that target and disrupt bacterial cellular membranes with compounds which neutralize bacterial virulence mechanisms while supporting the host body’s immune responses it should be possible to completely neutralize infective bacteria. This kind of multi-targeted approach, which is also common in conventional multidrug chemotherapy, should allow for prolonged treatment periods where necessary, while avoiding the ability of a specific pathogen to adapt and develop resistance to treatment, thereby providing relief from chronic conditions.

The kind of studies that already exist at this point, indicate that it should be possible to develop novel plant based topical formulations for ocular trachoma and that preliminary clinical studies could be performed in the near future with little more effort [9,42,43]. To continue to develop a range of standardized modern treatments, a series of systematic experiments should be performed on formulations which have been shown to be effective against chlamydiae so as to identify specific compounds which are primarily responsible for the observed antichlamydial activity. Perhaps the greatest research challenge in this field is related to the probability that the observed activity of these formulations is related to the synergistic action of more than one compound within the formulation. In dealing with this issue it becomes important to consider the significance of studying the combinations of compounds present in particularly active biological materials so as to obtain insight into recombining specific compounds after their extraction or synthesis. Finally, to develop an effective treatment it is important to ensure that active compounds are successfully delivered to infection sites. In relation to the various compounds covered in this review, topical applications are likely to provide the most effective administration route. This is in line with the standard trachoma treatment modality of using topical tetracycline eye ointment [19,20].

In terms of the treatment and prevention of trachoma, it is important to consider the factor of *C. trachomatis* being a commonly sexually transmitted [9] and often undetected pathogenic bacteria [12]. The high rates of infection along with high rates of transmission from mother to infant [14] are clearly a major source of new ocular trachoma infections in children. Even when the disease does not manifest soon after birth, it is known that *C. trachomatis* may remain largely dormant and slowly manifest over many years [4] especially as the host’s immune system becomes compromised by other factors. The study and treatment of ocular and vaginal chlamydial infections should be seen as a common goal and this raises the importance as well as the benefits of the eradication of these diseases even more. Developing treatments for one condition will contribute significantly to advancing the treatment of the other. Successfully dealing with trachoma, the most common cause of blindness in the world, as well as dealing with genital chlamydial infections, the most common sexually transmitted bacterial pathogen, would be a significant achievement in the fields of both medicine and human health.
